# Quinia as a Parturient

**Published:** 1875-02

**Authors:** B. H. Washington

**Affiliations:** Augusta, Georgia


### QUINIA AS A PARTURIENT.

BY B. H. WASHINGTON, M.D., OF AUGUSTA, GEORGIA.

Allow me to call attention to a few thoughts which may serve to put in the proper light the administration of quinine in pregnancy. Any one acquainted with the medical practice in vogue some thirty years ago, knows very well that quinine was freely administered to pregnant women, and sometimes administered immediately previous to delivery, to prevent the complications of a chill with those of delivery, and no one ever saw any labor pains brought on by quinine. On the other hand, observers, perfectly reliable, affirm that they have seen labor pains increased by the use of quinine.

Dry-cupping to the spinal column will cause the relaxation of the os uteri and contraction of the fundus uteri, and all at the same time; furthermore, in cases of habitual miscarriage, no remedy will more effectually prevent it, and yet, when the time for labor comes on, it will make more efficient the very pains it had been holding back for four or five months.

I took charge of a case once, in her seventh pregnancy, about to miscarry at the very same time she had done for six preceding pregnancies. The pains were very severe, regular and efficient. I gave a heavy opiate, and applied a small blister to the spine, and while she was sleeping the blister was at work, breaking up the morbid condition of the nervous system. About the third or fourth day after, regular dry-cupping the whole length of the spinal column was tried every alternate night during the remainder of her pregnancy, and she went to her full time and was safely and easily delivered. And yet, in tedious labors, it is one of the most efficient agents we have for rectifying affairs.

As I mentioned not many months since, Professor E. D. Fenner reported a case in labor thirty hours, when he concluded to try my plan, and she was safely delivered in thirty minutes.

As this affirmation, that dry-cupping will produce expansion of the os uteri and contraction of the fundus, seems to be a puzzle to many, and very unreasonable, allow me to state how I was convinced, for you have many new readers since my first articles, and as logicians say it is the best plan to use the same reasons to convince others that convinced you, I will give the reasons which convinced me.

First, then, I dry-cupped a lady on the second night after her confinement, to relieve the disagreeable results usually following, and she remarked that it made her uterus contract.

Some time after, I had myself dry-cupped, and while the cup was high up on the neck, I attempted to drive away some insect that was stinging me on the cheek, and found my arm hang very heavy. The thought immediately occurred to me that dry-cupping was just the very thing to take off muscular resistance in dislocation of the humerus.

Some months afterward, a boy was thrown from a horse, and had a dislocation at the shoulder-joint. I tried the dry-cupping, and it took off the muscular resistance so effectually that the boy scarcely made a wry face when the bone was replaced.

Here, then, was something to reason upon: the woman affirmed that dry-cupping made her uterus *contract*, and it was very clear that, in this case of the boy, dry-cupping had produced *relaxation*—both facts which could not be disputed. Why, then, I asked myself, will not dry-cupping produce contraction of the fundus uteri and expansion of the os, inasmuch as the proper function of the first is to contract, and of the second to expand?

Upon the first trial, in a case of tedious labor, the woman was safely delivered in less than thirty minutes, and in various other cases in less than fifteen minutes.

Here, then, we have a remedy which will cause prompt and efficient uterine action at the right time, and ten days before the right time will not cause action.

Now let us revert to quinine, and we may safely say that, up to the right time, quinine may be administered without any calculations of its causing uterine contractions, and yet, at the right time, it may be considered an efficient adjuvant in natural labor, or even efficient in those cases where the threatened miscarriage has proceeded to such a stage as to be utterly beyond the reach of art to check. In one case I had, quinine was given in four or five-grain doses every three hours, every day for ten or fifteen days, to crush down a remittent fever, antecedent to her delivery, and this was accomplished; the woman went on to her calculated time, not missing her set time ten hours, and was delivered without any remittent complications, and without any disturbance from the quinine.

In conclusion, then, I must say that quinine seems to have no ecbolic or parturient effects until the allotted time has arrived, and then it may be useful as an adjuvant in causing uterine pains—this, however, I must say on the authority of others, for I have never seen any parturient effects antecedent to the proper labor, and have never used it in labors.

My desire is to place the subject in such a light that no woman will be allowed to suffer from any intermittent or remittent complications anterior to her normal labor, through the fear of her attendant that quinine may prove such an ecbolic as to cause her to miscarry.—*Nashville Journal of Medicine and Surgery.*

---

Electricity in Amenorrhœa.—Amenorrhœa occurring in women otherwise healthy is treated by Dr. Althaus with the constant galvanic current applied as follows: The negative pole of a Daniell's battery of fifty to sixty elements is placed alternately over the right and left ovarian region; the positive over the lumbar vertebræ or the os uteri. Each sitting lasts fifteen minutes—*Medical Times and Gazette.*

